# Supplementary material for: Bayesian refinement of protein structures and ensembles against SAXS data using molecular dynamics
Source: PLoS Comput Biol. 2017 Oct 18;13(10):e1005800. doi: 10.1371/journal.pcbi.1005800 (PMC5662244; doi:10.1371/journal.pcbi.1005800)
Supplement: S1 Archive — (BZ2) [file pcbi.1005800.s011.tar.bz2 › gromacs_4.62_bayesian_saxs_refi/share/html/online/yourown.html]

Getting started - Your Own


| Getting started - Your Own Main Table of Contents |

**VERSION 4.6  
Sat 19 Jan 2013**

---

## Your own System

For proteins in water (or other solvent) the route is described above.
For other systemd (eg. pure liquids or mixtures) one needs:

- The atomic coordinates, which can be generated by a variety of
  interactive programs (eg. Quanta, Cerius, HyperChem).
  Coordinate files can be exported in pdb-format and
  converted to .gro format by
  the editconf program:
    
    

  |  |  |  |
  | --- | --- | --- |
  |  | editconf -f conf.pdb -o conf.gro   \| |

    
  where conf.gro is the coordinatefile,
  or converted back to pdb-format by
    
    

  |  |  |  |
  | --- | --- | --- |
  |  | editconf -f conf.gro -o conf.pdb   \| |

    
  where conf is a file with coordinates, and 
  conf.pdb is the target file in .pdb format.
  **NOTE:** Make sure that the graphics programs export
  **whole** molecules instead of molecules that are cut in pieces
  (due to the periodic boundary conditions)
  If you have the coordinates of single molecules, you can also
  build systems (pure liquids or mixtures) with
  genbox.
  In contrast, the program
  genconf
  produces the lattice of molecules with random displacements.- The topology you have to build yourself. Of course you can
    include topologies of part of your system (eg. spc.itp,
    decane.itp etc.)

---


### Back to start


---
